# Increased Blood Retinol Levels Are Associated With a Reduced Risk of TIA or Stroke in an Adult Population: Lifestyle Factors- and CVDs-Stratified Analysis

**DOI:** 10.3389/fcvm.2021.744611

**Published:** 2021-11-16

**Authors:** Linjuan Guo, Ying Huang, Rong Wan, Yang Shen, Kui Hong

**Affiliations:** ^1^Department of Cardiovascular Medicine, The Second Affiliated Hospital of Nanchang University, Nanchang, China; ^2^Jiangxi Key Laboratory of Molecular Medicine, Nanchang, China

**Keywords:** blood retinol, stroke, transient ischemic attack, lifestyle factors, cardiovascular diseases

## Abstract

**Background:** Data on the existing evidence for the association between blood retinol and transient ischemic attack (TIA)/stroke risk are limited, and the results are inconclusive. This study aimed to further assess the associations between the blood retinol levels and the risk of TIA/stroke after controlling the lifestyle factors and age-related confounders.

**Methods:** The cross-sectional data from 1,113 individuals (aged 34–84 years old) were obtained from the Midlife in the United States (MIDUS) study. The multivariable analyses were performed to investigate the association of blood retinol levels with ever and currently TIA/stroke.

**Results:** There was an inverse association between the blood retinol levels and the risk of ever TIA or stroke (for per 1 μmol/L adjusted odds ration [*OR*]: 0.93; 95% *CI*: 0.89–0.97; for per 1-SD adjusted *OR*: 0.89; 95% *CI*: 0.83–0.96) and currently diagnosed TIA or stroke (for per 1 μmol/L adjusted *OR*: 0.91; 95% *CI*: 0.87–0.96; for per 1-SD adjusted *OR*: 0.84; 95% *CI*: 0.80–0.91) after controlling the lifestyle factors and age-related confounders. The significance of these associations was maintained after a sensitivity analysis and involving “ever chronic respiratory diseases” as a covariate. Moreover, the stratified analyses suggested that the inverse associations could be affected by overweight [body mass index (BMI) ≥ 28, kg/m^2^], hypertension, and diabetes.

**Conclusions:** A significant inverse association between blood retinol and the risk of TIA or stroke was found. This inverse association did not change even after adjustment for many potential confounders. Moreover, the potential protective effect of retinol on TIA/stroke could be blunted by overweight [BMI ≥ 28, kg/m^2^], hypertension, and diabetes.

## Introduction

A transient ischemic attack (TIA) or stroke is a global public health problem that leads to the second leading cause of death (1). However, to date, there are few practical measures to prevent the TIA/stroke. The knowledge of risk factors is an essential step in predicting and further preventing the TIA/stroke. The existing evidence has suggested that the risk factors for TIA/stroke mainly include obesity, lack of physical exercise, hyperlipidemia, smoking, drinking, hypertension, diabetes, heart disease, or others (2). Importantly, in the recent years, the effect of nutrients on the TIA/stroke risk has become the focus of scientific discussion. The guidelines of the American Heart Association (AHA) and American Stroke Association (ASA) recommend that the diets low in saturated fats and salt, and rich in fiber, fruits, and vegetables can effectively reduce the occurrence of TIA/stroke (3).

Blood retinol is a lipid-soluble micronutrient involved in the processes of vision, cell differentiation and proliferation, and immune system regulation (4). In addition, the emerging evidence suggests that retinol has an important inhibitory effect on thrombosis and may also participate in an endothelial function by regulating the nitric oxide pathway (5, 6). A study found that blood retinol can regulate the chronic inflammation and stabilize plaque (7). It is well known that the chronic inflammation and plaque formation are the critical processes in developing atherosclerosis and TIA/ stroke. However, data on the association between blood retinol and TIA/stroke risk were limited in the prospective research, and the results were inconclusive (8, 9). More importantly, possible modifiers that impact the relationship between blood retinol and the risk of TIA/stroke are ambiguous.

To our knowledge, few previous studies have comprehensively examined the relationship between blood retinol and the risk of TIA/stroke. The Midlife in the United States (MIDUS) study, a large population-based cohort study of adult individuals (aged 34–84 years) that includes detailed information on the levels of blood nutrients, incidence of cardiovascular diseases (CVDs), and potential confounders, such as the lifestyle factors and age-related confounders. Hence, this study aimed to analyze the impact of blood retinol on TIA/stroke and identify the modifying factors of this impact.

## Methods

**Figure 1:**
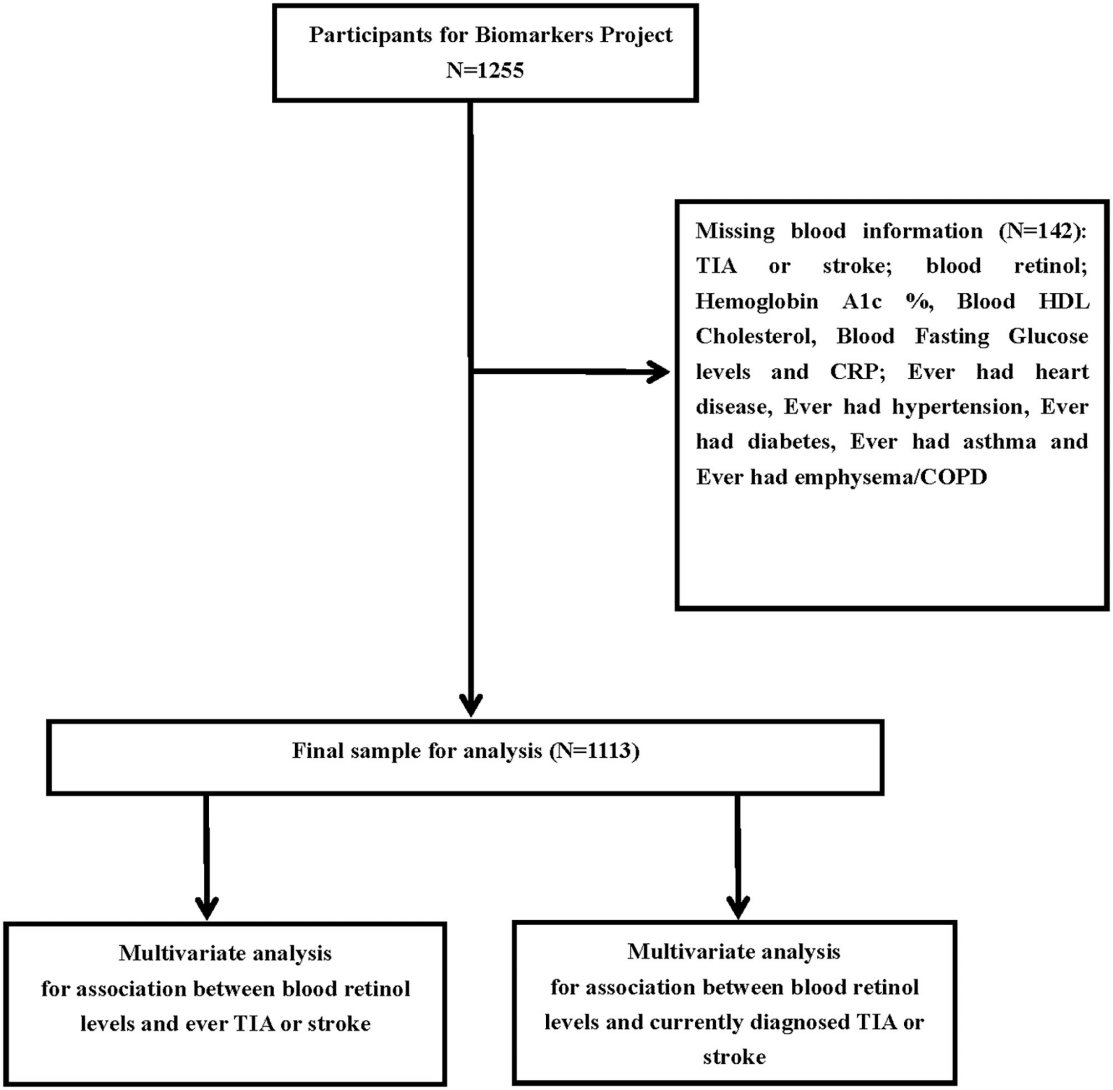
A detailed flow chart of subjects included in the analysis.

This study contains 1,113 subjects from the MIDUS II study, which is a 9-year follow-up of the MIDUS I cohort from 2004 to 2006, such as the demographic, behavioral, and social factors of an adult American population (10). The well-trained staff evaluated a self-administered survey of a wide array of behavioral, social, and psychological factors, and conducted the collection of biomarker project and data during a 2-day visit to the Clinical Research Center (CRC) at the University of California-Los Angeles, Wisconsin, or Georgetown. The respondents completed a list of chronic diseases and asked if they had experienced or been diagnosed with a list of chronic health conditions in the past 12 months, such as stroke/TIA. Blood data was collected from 2004 to 2009 (11). Full details of the MIDUS study biomarker protocol were available elsewhere (12, 13). Additionally, the fasting blood samples of the subjects were sent to the MIDUS Biocore laboratory for the analysis, and the blood levels, such as retinol, hemoglobin A1c, homeostasis model assessment of insulin resistance (HOMA-IR), fasting blood glucose, fasting insulin, total cholesterol, triglycerides, low-density lipoprotein (LDL)-cholesterol, high-density lipoprotein (HDL)-cholesterol, and C-reactive protein (CRP) were determined. The complete data and specific codebooks are provided at http://www.midus.wisc.edu/. After excluding the missing values, we included 1,113 participants for analysis, as shown in Figure 1. This study followed the guidelines of the Declaration of Helsinki, of which the ethics committee of each clinical research center approved data collection at the three locations and all the study participants signed the informed consent.

## Statistical Analysis

In this study, SPSS 26.0 (IBM, NY, USA) was used to perform all the analyses. All continuous variables were non-normally distributed, we represented them by using the median (interquartile range [IQR]) and standardized them by using the *Z*-score. Then, a multivariate logistic regression analysis was performed. The significance levels were corrected in each model to account for the relationships among the blood retinol levels and ever TIA/stroke and currently diagnosed TIA/stroke, respectively. No adjustment was made in a Crude Model. In Model 1, the blood retinol levels were regressed on both ever TIA/stroke and currently diagnosed TIA/stroke after adjusting for age, gender, and marital status. In Model 2, hemoglobin A1c %, HOMA-IR, fasting glucose levels, fasting insulin levels, total cholesterol, triglycerides, HDL cholesterol, LDL cholesterol, creatinine, and CRP were further adjusted. The categorical variables were defined as *n* (%). The subjects with missing data for specific variables were removed from the analysis.

In addition, we then used “chronic respiratory diseases (asthma and emphysema/COPD)” as a covariate for sensitivity analysis to further clarify the impact on blood retinol and its relationship with TIA/stroke. Finally, we performed the stratified analyses using the covariates, such as “overweight [body mass index (BMI) ≥ 28, kg/m^2^],” “current smoking,” “number of years of drinking (≥6),” “exercise frequency ≥3/week,” “heart disease,” “hypertension,” and “diabetes”. These stratified variables were used to examine whether the lifestyle factors and CVDs affect the association between the blood retinol levels and TIA/stroke. A *P-*value ≤ 0.05 was considered statistically significant.

## Results

### Characteristics of the Participants Stratified by Using the Blood Retinol Levels (*N* = 1,113)

Due to the lack of complete data for 142 participants, data for the remaining 113 participants were included in the study and further analyzed. Table 1 lists the characteristics of all the included individuals. The median blood retinol of this cohort study was 1.66 μmol/L. Based on the median blood retinol level, we divided the entire study cohort into two groups (Retinol <1.66 and ≥1.66 μmol/L). Comparison of the effect factors between the two groups, there were significant differences in BMI, current smoking status, number of drinking years, frequency of exercises ≥3/week, currently diagnosed TIA/stroke, and ever TIA/stroke, while there were no significant differences in age, gender, and marital status married. In terms of the CVDs history (heart disease, hypertension, and diabetes), individuals with the blood retinol levels below the median were more common than those with the blood retinol levels at or above the median. For the chronic respiratory diseases, emphysema/COPD was more prevalent in the individuals with lower-level blood retinol. Moreover, hemoglobin A1c, HOMA-IR, fasting glucose levels, fasting insulin levels, total cholesterol, LDL cholesterol, CRP were lower, while the HDL cholesterol was higher in the individuals with the higher blood retinol levels. There were no significant differences in the triglyceride and creatinine levels between the two groups.

**Table 1:** Characteristics of participants stratified by blood retinol levels (*N* = 1,113).

**Variables**	**Total participants (*N* = 1,113)**	**Retinol < 1.66 umol/L (*N* = 557)**	**Retinol ≥1.66 umol/L (*N* = 556)**	***P*-value**
Age (years)	53.0 (45.0–62.0)	52.5 (44.6–61.3)	53.2 (45.6–62.4)	0.112
Gender (male), *n* (%)	470 (43.2)	251 (45.1)	219 (39.4)	0.051
Currently married, *n* (%)	688 (61.8)	354 (63.6)	334 (60.1)	0.392
BMI (kg/m^2^)	28.5 (25.2–32.9)	30.7 (27.3–35.2)	27.1 (22.8–30.5)	<0.001
Current smoker, *n* (%)	168 (15.1)	108 (19.4)	60 (10.8)	<0.001
Number of drinking years	7.0 (3.0–23.0)	8.0 (4.0–24.0)	5.0 (2.0–21.0)	0.021
Frequency of exercises ≥ 3/week, *n* (%)	821 (73.8)	391 (70.2)	430 (77.3)	0.010
Currently diagnosed TIA or stroke, *n* (%)	54 (4.9)	38 (6.8)	16 (2.9)	<0.001
Ever TIA /stroke, *n* (%)	31 (2.7)	20 (3.5)	11 (2.0)	0.002
**CVDs history**				
Heart disease, *n* (%)	112 (10.1)	75 (13.5)	37 (6.7)	0.011
Hypertension, *n* (%)	422 (37.9)	265 (47.6)	157 (28.2)	<0.001
Diabetes, *n* (%)	102 (9.2)	65 (11.7)	37 (6.7)	0.026
**Ever chronic respiratory diseases**				
Asthma, *n* (%)	129 (11.6)	71 (12.7)	58 (10.4)	0.267
Emphysema/COPD, *n* (%)	39 (3.5)	31 (5.6)	8 (1.4)	0.002
**Blood analysis**				
Hemoglobin A1c %	5.7 (5.3–6.1)	6.2 (5.4–7.3)	5.3 (5.0–6.5)	0.018
HOMA-IR	2.4 (1.5–4.4)	2.6 (1.7–4.9)	2.1 (1.2–4.1)	0.020
Fasting glucose levels mg/Dl	95.0 (92.0–108.0)	98.0 (94.0–112.0)	92.0 (89.0–101.0)	0.012
Fasting insulin levels uIU/mL	11.0 (5.0–16.0)	13.0 (6.0–18.0)	9.0 (3.0–15.0)	0.017
Total Cholesterol (mg/dL)	182.7 (162.1–219.3)	189.5 (166.3–224.8)	180.1 (160.3–213.5)	0.004
Triglycerides (mg/dL)	105.3 (76.3–154.7)	105.9 (76.6–156.3)	105.1 (75.8–153.8)	0.065
HDL Cholesterol (mg/dL)	54.3 (44.6–67.2)	52.6 (42.7–63.8)	55.1 (45.3–68.5)	0.025
LDL Cholesterol (mg/dL)	124.8 (81.6–128.8)	135.6 (87.9–148.6)	116.7 (79.6–119.3)	0.003
Creatinine (mg/dL)	0.85 (0.72–1.46)	0.91 (0.73–1.59)	0.83 (0.70–1.35)	0.071
C-reactive protein (ug/mL)	1.34 (0.62–3.36)	1.81 (0.76–3.99)	1.27 (0.57–3.2)	0.013

*M (IQR) for non-normally distributed variables, and n (%) for categoric variables*.

*BMI, Body mass index; TIA, transient ischemic attack; CVDs, Cardiovascular diseases; COPD, chronic obstructive pulmonary disease; HOMA-IR, homeostasis model assessment of insulin resistance; HDL, high density lipoprotein; LDL, low density lipoprotein; CRP, C-reactive protein*.

### Increased Blood Retinol Levels Were Associated With a Reduced Risk of TIA/Stroke by Using the Multivariate Logistic Regression Analysis

The results of the multivariate logistic regression analysis for the association between the blood retinol levels, and ever and currently diagnosed TIA/stroke are shown in Table 2. In the Crude model (unadjusted), with the increment of blood retinol per 1 μmol/L, the incidence of ever TIA/stroke decreased 10% (odds ratio [*OR*]: 0.90; 95% *CI*: 0.84–0.95) and currently diagnosed TIA/stroke decreased 13% (*OR*: 0.87; 95% *CI*: 0.79–0.92). Similarly, with the increment of blood retinol per 1-SD increase, the incidence of ever TIA/stroke decreased 17% (*OR*: 0.83; 95% *CI*: 0.75–0.90), and currently diagnosed TIA/stroke decreased 20% (*OR*: 0.80; 95% *CI*: 0.70–0.86). When adjusted for model 1 (adjustment for the confounding factors potentially associated with TIA or stroke), as the blood retinol increased per 1 μmol/L, the risk of ever TIA/stroke was reduced by 9% (*OR*: 0.91; 95% *CI*: 0.86–0.96) and currently diagnosed TIA/stroke was reduced by 10% (*OR*: 0.90; 95% *CI*: 0.85–0.94). Likewise, with the blood retinol increased every 1-SD, the risk of ever TIA/stroke was reduced by 15% (*OR*: 0.85; 95% *CI*: 0.77–0.91) and currently diagnosed TIA/stroke was reduced by 18% (*OR*: 0.82; 95% *CI*: 0.74–0.90). In model 2, with the increment of blood retinol per 1 μmol/L, the incidence of ever TIA/stroke decreased by 7% (*OR*: 0.93; 95% *CI*: 0.89–0.97), and currently diagnosed TIA/stroke decreased by 9% (*OR*: 0.91; 95% *CI*: 0.87–0.96). The incidence of ever had TIA/stroke decreased by 11% (*OR*: 0.89; 95% *CI*: 0.83–0.96) and currently diagnosed TIA or stroke decreased by 16% (*OR*: 0.84; 95% *CI*: 0.80–0.91) when the blood retinol increased per 1-SD.

**Table 2:** Multivariate logistic regression analysis for association between blood retinol levels and TIA /stroke (*N* = 1,113).

**Blood retinol (umol/L)**	**Ever TIA /stroke**			**Currently diagnosed TIA /stroke**		
	***OR***	**95% CI**	***P*-value**	***OR***	**95% CI**	***P*-value**
**Crude**						
Continuous, per 1umol/L increment	0.90	0.84–0.95	0.003	0.87	0.79–0.92	<0.001
Per 1-SD increase	0.83	0.75–0.90	<0.001	0.80	0.70–0.86	<0.001
**Model 1**						
Continuous, per 1umol/L increment	0.91	0.86–0.96	0.012	0.90	0.85–0.94	<0.001
Per 1-SD increase	0.85	0.77–0.91	<0.001	0.82	0.74–0.90	<0.001
**Model 2**						
Continuous, per 1umol/L increment	0.93	0.89–0.97	0.024	0.91	0.87–0.96	<0.001
Per 1-SD increase	0.89	0.83–0.96	0.002	0.84	0.80–0.91	<0.001

*Crude: Adjusted for no*.

*Model 1: Adjusted for age, gender and marital status*.

*Model 2: Adjusted for age, gender, marital status, blood analysis including hemoglobin A1c %, HOMA-IR, fasting glucose levels, fasting insulin levels, total cholesterol, triglycerides, HDL cholesterol, LDL cholesterol, creatinine and C-reactive protein*.

*TIA, transient ischemic attack; HOMA-IR, homeostasis model assessment of insulin resistance; HDL, high density lipoprotein; LDL, low density lipoprotein*.

### Increased Blood Retinol Levels Contributed to a Reduced Risk of TIA/Stroke by Using the Sensitivity Analysis

The sensitivity analyses (Table 3) showed that the *OR*s were little changed by further adjustment for “ever chronic respiratory diseases” in the Crude Model, as well as in the model 1 and model 2 (all *P* < 0.05). These results further suggested that the significant and independent associations between the blood retinol levels and ever TIA/stroke and currently diagnosed TIA/stroke.

**Table 3:** Sensitivity analysis for association between blood retinol levels and TIA /stroke (*N* = 1,113).

**Blood retinol (umol/L)**	**Ever TIA /stroke**			**Currently diagnosed TIA /stroke**		
	***OR***	**95% CI**	***P*-value**	***OR***	**95% CI**	***P*-value**
**Crude**						
Continuous, per 1umol/L increment	0.91	0.83–0.96	0.004	0.86	0.78–0.91	<0.001
Per 1-SD increase	0.85	0.78–0.93	0.001	0.82	0.74–0.87	<0.001
**Model 1**						
Continuous, per 1umol/L increment	0.92	0.87–0.97	0.016	0.90	0.84–0.95	<0.001
Per 1-SD increase	0.87	0.79–0.94	0.005	0.85	0.77–0.93	<0.001
**Model 2**						
Continuous, per 1umol/L increment	0.92	0.89–0.98	0.031	0.92	0.88–0.97	<0.001
Per 1-SD increase	0.88	0.86–0.97	0.009	0.86	0.84–0.992	<0.001

*Crude: Adjusted for ever chronic respiratory diseases*.

*Model 1: Adjusted for age, gender, marital status and ever chronic respiratory diseases*.

*Model 2: Adjusted for age, gender, marital status, blood analysis including hemoglobin A1c %, HOMA-IR, fasting glucose levels, fasting insulin levels, total cholesterol, triglycerides, HDL cholesterol, LDL cholesterol, creatinine and C-reactive protein and ever chronic respiratory diseases*.

*TIA, transient ischemic attack; HOMA-IR, homeostasis model assessment of insulin resistance; HDL, high density lipoprotein; LDL, low density lipoprotein*.

We additionally performed the stratified analyses to assess the effect of blood retinol (per 1 μmol/L and 1-SD increment) on ever TIA/stroke and currently diagnosed TIA/stroke by adding several factors of lifestyle and CVDs as a covariate, respectively (Table 4). Our results suggested that with the blood retinol increase by 1 μmol/L or 1-SD, the risk of ever TIA/stroke and currently diagnosed stroke/TIA were still decreased in the individuals with no-overweight, no-hypertension, and no-diabetes (all *P* < 0.05), but not decreased in the individuals with the three covariates (all *P* > 0.05). However, our results showed that the association between the blood retinol and TIA/stroke were not affected by the current smoking status, number of drinking years, frequency of exercises ≥3/week, and heart disease. We first found that overweight, hypertension, and diabetes were modifiers that affect the association between blood retinol and ever TIA/stroke and currently diagnosed stroke/TIA.

**Table 4:** Stratified analysis for association between blood retinol levels and TIA /stroke using multivariate logistic regression models (*N* = 1,113).

	**Ever TIA /stroke**			**Currently diagnosed TIA/stroke**		
**Blood retinol (umol/L)**	***OR***	**95% CI**	***P*-value**	***OR***	**95% CI**	***P*-value**
**BMI (≥28, kg/m** ^**2**^ **)**						
Continuous, per 1 umol/L increment	0.97	0.92–1.22	0.235	0.94	0.90–1.16	0.093
Per 1-SD increase	0.95	0.90–1.20	0.194	0.93	0.88–1.14	0.089
**BMI (<28, kg/m** ^**2**^ **)**						
Continuous, per 1 umol/L increment	0.69	0.48–0.85	0.007	0.61	0.42–0.80	<0.001
Per 1-SD increase	0.62	0.44–0.83	0.001	0.55	0.38–0.72	<0.001
**Current smoking (yes)**						
Continuous, per 1 umol/L increment	0.94	0.89–0.99	0.029	0.92	0.88–0.94	0.003
Per 1-SD increase	0.85	0.80–0.92	0.010	0.82	0.82–0.94	<0.001
**Current smoking (no)**						
Continuous, per 1 umol/L increment	0.88	0.82–0.94	0.013	0.84	0.79–0.91	<0.001
Per 1-SD increase	0.83	0.75–0.91	0.009	0.80	0.71–0.86	<0.001
**Number of drinking years (≥6)**						
Continuous, per 1 umol/L increment	0.92	0.87–0.96	0.030	0.89	0.82–0.95	0.002
Per 1-SD increase	0.83	0.78–0.90	0.012	0.80	0.78–0.92	<0.001
**Number of drinking years (<6)**						
Continuous, per 1 umol/L increment	0.89	0.83–0.95	0.015	0.86	0.81–0.94	<0.001
Per 1-SD increase	0.84	0.77–0.91	0.013	0.81	0.76–0.92	<0.001
**Frequency of exercises ≥3/week (yes)**						
Continuous, per 1 umol/L increment	0.90	0.84–0.95	0.012	0.86	0.78–0.91	0.001
Per 1-SD increase	0.81	0.73–0.87	0.008	0.75	0.62–0.81	<0.001
**Frequency of exercises ≥3/week (no)**						
Continuous, per 1 umol/L increment	0.91	0.84–0.97	0.017	0.87	0.82–0.94	<0.001
Per 1-SD increase	0.86	0.79–0.95	0.015	0.83	0.78–0.93	<0.001
**Heart disease (yes)**						
Continuous, per 1 umol/L increment	0.87	0.78–0.94	0.008	0.82	0.71–0.83	<0.001
Per 1-SD increase	0.72	0.61–0.83	0.001	0.70	0.59–0.82	<0.001
**Heart disease (no)**						
Continuous, per 1 umol/L increment	0.92	0.86–0.95	0.007	0.84	0.72–0.84	<0.001
Per 1-SD increase	0.81	0.70–0.82	0.018	0.73	0.67–0.80	<0.001
**Hypertension (yes)**						
Continuous, per 1 umol/L increment	0.96	0.90–1.19	0.232	0.92	0.89–1.14	0.092
Per 1-SD increase	0.95	0.88–1.17	0.197	0.90	0.87–1.13	0.087
**Hypertension (no)**						
Continuous, per 1 umol/L increment	0.65	0.45–0.83	0.005	0.59	0.40–0.76	<0.001
Per 1-SD increase	0.61	0.43–0.86	<0.001	0.56	0.41–0.73	<0.001
**Diabetes (yes)**						
Continuous, per 1 umol/L increment	0.94	0.85–1.17	0.165	0.88	0.78–1.10	0.076
Per 1-SD increase	0.90	0.81–1.14	0.088	0.82	0.71–1.03	0.068
**Diabetes (no)**						
Continuous, per 1 umol/L increment	0.68	0.47–0.84	0.008	0.62	0.42–0.78	<0.001
Per 1-SD increase	0.62	0.42–0.80	0.003	0.58	0.39–0.70	<0.001

*Adjusted for age, gender, marital status, blood analysis including hemoglobin A1c %, HOMA-IR, fasting glucose levels, fasting insulin levels, total cholesterol, triglycerides, HDL cholesterol, LDL cholesterol, creatinine and C-reactive protein*.

*TIA, transient ischemic attack; BMI, Body mass index; HOMA-IR, homeostasis model assessment of insulin resistance; HDL, high density lipoprotein; LDL, low density lipoprotein*.

## Discussion

We found that the increased blood retinol levels were associated with a reduced risk of TIA/stroke. This dependent association did change little after the adjustments for many lifestyle factors and age-related confounders. The potential protective effect of retinol on TIA/stroke can be attenuated by overweight, hypertension, and diabetes.

As early as 1992, it was reported that the patients with ischemic stroke with the blood retinol concentrations above 2.27 μmol/L had increased rates of recovery and decreased mortality (14). In contrast, a prospective cohort study, which enrolled 1,031 patients in Finland, with an average follow-up of 12.1 years, found that in patients with a high concentration of blood retinol ≥68 μg/dl compared with those with a low concentration of blood retinol <51 μg/dl, the risk of stroke was not reduced (9). However, this study only included 67 stroke events, and there were not enough samples to assess the association between blood retinol and stroke. Similarly, a nested case-control study consisting of 297 ischemic stroke cases and 297 controls, with an average follow-up of 7.3 years was conducted. The results of this study suggested that there was no association between blood retinol and the risk of ischemic stroke, but the study was only conducted in a male population (8). These previous studies, which have inconsistent results, did not include enough samples and did not explore the relationship between retinol and stroke risk in the general population. Additionally, these inconsistent results may be caused by the difference in the age-related confounding factors, such as, the populations selected, the use of medications, and varying blood biochemical examinations. Therefore, it is necessary to further study whether the increased blood retinol levels can contribute to a lower risk of stroke after excluding these limitations.

Our study found a significant inverse association between blood retinol with the risk of ever TIA/stroke and currently diagnosed stroke/TIA in the general population with a large sample. The inverse association was mainly found in the participants with BMI < 28, non-hypertension, and non-diabetes. Espe et al. reported a study that included 1,177 patients with diabetic hemodialysis with an average follow-up of 4 years. Compared with the patients with diabetic hemodialysis with blood retinol ≥111 μg/dl, the patients with blood retinol 91–111 μg/dl did not have a reduced risk of stroke, while the risk did not increase in the patients with blood retinol <74 μg/dl (15). This result is consistent with the association between blood retinol and the risk of stroke in the patients with diabetes in our study. However, the pathophysiology of the patients with diabetes was different from that of the general population. Therefore, we then conducted a stratified analysis and found that diabetes was a modifier factor in the association between blood retinol and stroke. In the patients with non-diabetes, blood retinol and stroke have a significant inverse association. One previous study has conducted a stratified analysis further to find the modifier factor between blood retinol and stroke. A total of 20,702 patients with hypertension were included in a recent China Stroke Primary Prevention Trial (CSPPT) study. The results of the study suggested a significant negative correlation between blood retinol and the first stroke (per 10-μg/dl increment; *OR*: 0.92; 95% *CI*: 0.86, 0.97) and first ischemic stroke (per 10-μg/dl increment; *OR*: 0.92; 95% *CI*: 0.86, 0.98), and homocysteine was a modifier factor for the association. However, the participants in this study were all patients with hypertension, and the results may not apply to the generality of other populations (16).

Our study was the first to thoroughly examine any possible modifiers that affect the association between blood retinol and TIA/stroke in the general people, and found that overweight, hypertension, and diabetes were the effective modifier factors. It is well known that overweight, hypertension, and diabetes were the independent and harmful risk factors for stroke (17). In-depth investigation of the mechanism of overweight, it was found that the chronic mild inflammation and altered intestinal microbes play an important role in obesity (18, 19), and adipose tissue was not only considered as an inert energy store but also secretes various adipokines (such as, leptin, adiponectin, and lipase) and cytokines [such as, tumor necrosis factor-alpha (TNF-α), interleukins (ILs), and monocyte chemotactic protein (MCP)]. These adipokines and cytokines are the primary mediators of inflammation and are related to obesity-related inflammatory complications, such as insulin resistance and hypertension (20, 21), which eventually increased the risk of stroke (22–24). Interestingly, the growing evidence has shown that blood retinol has a potential protective effect on obesity, diabetes, and hypertension (25, 26). Zulet et al. reported a negative relationship between the blood retinol intake and obesity (27). Other studies have reported an inverse relationship between blood retinol and BMI in the morbidly obese subjects (28–32). Additionally, blood retinol and hypertension have a negative relationship. Multiple studies on the individuals with hypertension found that the patients with hypertension have a low blood retinol level (33, 34). Retinol acyltransferase (LRAT) is a key enzyme for converting and storing retinol in the tissues. The studies have found that the level of LRAT was negatively correlated with blood pressure, and the LRAT of the hypertension group was lower than that of the non-hypertension group (35). The metabolic syndrome (METS) refers to a group of risk factors that are highly related to metabolism. The specific classification criteria of METS are not yet unified, but it is generally believed that METS includes diabetes, dyslipidemia, obesity, and hypertension. Recently, a study on patients with METS found that the patients with METS have a significantly lower level of blood retinol than the normal control group (36). Therefore, it seems that the harmful effects of diabetes, obesity, and hypertension on stroke may be weakened by the potential protective effect of retinol. These results might also further explain our finding that a strong negative correlation between blood retinol and stroke was found in the people with non-diabetic and non-hypertensive, BMI < 28. Our results also emphasize that maintaining the higher blood retinol levels may play an important role in preventing TIA/stroke after controlling overweight, hypertension, and diabetes.

There were also some limitations in this study. First, dynamic measurement of the blood retinol levels can more accurately assess the association between blood retinol and TIA/stroke risk. In this study, the blood retinol levels of participants were only evaluated in a cross-section of time. Therefore, we were unable to assess whether the blood retinol changes have an impact on the incidence of TIA/stroke. Second, although the main potential confounding factors were controlled, the results may be affected by the unmeasured or uncertain confounding factors. Third, we did not have detailed dietary information about the source of retinol for the participants. Retinol can be obtained from the animal foods, such as milk, red meat, offal, and cheese, and can also be obtained from the plant foods, such as carrots and corn. Whether retinol obtained from different source has different effects on TIA/stroke is not clear and should be further examined in future studies. Finally, stroke includes ischemic and hemorrhagic stroke, therefore, this study was insufficient in assessing the association between the blood retinol levels and specific subtypes of stroke. Further studies should be undertaken to investigate the prevalence of ischemic stroke/hemorrhagic stroke/TIA with the blood retinol levels.

## Conclusions

Although more research is needed, our data suggested that a healthy diet, such as appropriate retinol may help to prevent TIA/stroke through the potential protective effects of retinol. Our study provided the first evidence that the increased blood retinol levels may benefit TIA/stroke in the general population and the potential protective effect of retinol on TIA/stroke might be blunted by overweight, hypertension, and diabetes.

## Data Availability Statement

Publicly available datasets were analyzed in this study. This data can be found at: http://www.midus.wisc.edu.

## Ethics Statement

The studies involving human participants were reviewed and approved by Clinical Research Center (CRC) at the University of California-Los Angeles, Wisconsin, or Georgetown. The patients/participants provided their written informed consent to participate in this study.

## Author Contributions

KH was incharge of the entire project and revised the draft of the manuscript. LG and YH analyzed the data. LG drafted the first version of the manuscript. All authors took part in the interpretation of the results and in the preparation of the final version of the manuscript.

## Conflict of Interest

The authors declare that the research was conducted in the absence of any commercial or financial relationships that could be construed as a potential conflict of interest.

## Publisher's Note

All claims expressed in this article are solely those of the authors and do not necessarily represent those of their affiliated organizations, or those of the publisher, the editors and the reviewers. Any product that may be evaluated in this article, or claim that may be made by its manufacturer, is not guaranteed or endorsed by the publisher.
